# Multi-Pathogen Surveillance of Acute Febrile Illness in Nigeria Using TaqMan Array Cards: Implementation Successes and Lessons Learned From the SAFIAN Study

**DOI:** 10.1093/cid/ciaf514

**Published:** 2025-11-20

**Authors:** Jean H Kim, Philippe Chebu, Richard Fayomade, Onyia Justus Ejike, Claire A Quiner, Vivian Kwaghe, Cyril Erameh, Femi Owolagba, Blessed Okhiria, Ikponmwosa Odia, Jacqueline Agbukor, Julius Oshiobugie Imoyera, Walter Mary Odion, Katherine Asman, Adamu Zigwai Ephraim, Osahogie Isaac Edeawe, Nankpah Godsave Vongdip, Victoria Orok, Oladimeji Damilare Matthew, Ephraim Ogbaini-Emovon, Blessing Amierhobhiye Obagho, Jay Osi Samuels, Emmanuel A Oga, Lauren P Courtney

**Affiliations:** Solutions, RTI International, Durham, North Carolina, USA; Laboratory Services, APIN Public Health Initiatives, Federal Capital Territory, Abuja, Nigeria; Laboratory Services, APIN Public Health Initiatives, Federal Capital Territory, Abuja, Nigeria; Internal Medicine, University of Abuja Teaching Hospital, Abuja, Nigeria; Solutions, RTI International, Durham, North Carolina, USA; Internal Medicine, University of Abuja Teaching Hospital, Abuja, Nigeria; Institute of Viral and Emergent Pathogens Control and Research, Irrua Specialist Teaching Hospital, Edo, Nigeria; Laboratory Services, APIN Public Health Initiatives, Federal Capital Territory, Abuja, Nigeria; Internal Medicine, University of Abuja Teaching Hospital, Abuja, Nigeria; Institute of Viral and Emergent Pathogens Control and Research, Irrua Specialist Teaching Hospital, Edo, Nigeria; Institute of Viral and Emergent Pathogens Control and Research, Irrua Specialist Teaching Hospital, Edo, Nigeria; Institute of Viral and Emergent Pathogens Control and Research, Irrua Specialist Teaching Hospital, Edo, Nigeria; Institute of Viral and Emergent Pathogens Control and Research, Irrua Specialist Teaching Hospital, Edo, Nigeria; Solutions, RTI International, Durham, North Carolina, USA; Solutions, RTI International, Durham, North Carolina, USA; Institute of Viral and Emergent Pathogens Control and Research, Irrua Specialist Teaching Hospital, Edo, Nigeria; Internal Medicine, University of Abuja Teaching Hospital, Abuja, Nigeria; Internal Medicine, University of Abuja Teaching Hospital, Abuja, Nigeria; Internal Medicine, University of Abuja Teaching Hospital, Abuja, Nigeria; Institute of Viral and Emergent Pathogens Control and Research, Irrua Specialist Teaching Hospital, Edo, Nigeria; Institute of Viral and Emergent Pathogens Control and Research, Irrua Specialist Teaching Hospital, Edo, Nigeria; Laboratory Services, APIN Public Health Initiatives, Federal Capital Territory, Abuja, Nigeria; Solutions, RTI International, Durham, North Carolina, USA; ClineEpi Partners, Columbia, Maryland, USA; Solutions, RTI International, Durham, North Carolina, USA

**Keywords:** AFI, TAC, lessons learned, mutipathogen surveillance, SAFIAN

## Abstract

Undiagnosed acute febrile illness (AFI) is a persistent challenge in Nigeria, where the presence of malaria is often presumed in the absence of timely and accurate diagnostic confirmation. To expand diagnostic capacity and identify a broader spectrum of AFI etiologies, the Surveillance of Acute Febrile Illness Aetiology in Nigeria (SAFIAN) study implemented the TaqMan Array Card (TAC) platform to test for 25 pathogens among febrile patients at 2 tertiary hospitals. This article summarizes operational lessons from the introduction of TACs along with key implementation components, including platform selection, procurement and shipment of specialized equipment, laboratory preparation, staff training, and quality control oversight. We also highlight several constraints, including procurement delays, sample contamination, assay underperformance, and procedural inefficiencies. Findings from the SAFIAN study reveal that successful integration of TACs requires strong site-level coordination, real-time data and supply tracking, cross-team collaboration, and sustained investment in infrastructure and workforce capacity to improve detection and response efforts in high-burden environments.

## Teaser

We implemented TaqMan Array Cards for multipathogen detection of acute febrile illnesses, summarizing key lessons learned. Challenges involved delays, contamination, and inefficiencies. Successful study implementation required coordination, real-time tracking, collaboration, and sustained investment in infrastructure and workforce.

In an increasingly interconnected world, timely and accurate disease surveillance is essential to detect emerging infectious disease threats, guide public health responses, and strengthen global health security. Timely surveillance is especially necessary in sub-Saharan Africa, where a diverse ecology and varying socioeconomic drivers create environments that are conducive to a wide range of diseases [1, 2], thus increasing the risk of undetected outbreaks with potentially far-reaching consequences.

Despite its importance, disease surveillance is often hindered by time, staffing, and cost constraints, which can limit the feasibility of sustained monitoring efforts, particularly in resource-limited settings. These challenges are further compounded by the need for well-trained staff and expensive laboratory infrastructure.

To effectively monitor and respond to the complex range of pathogens causing acute febrile illness (AFI), there is an urgent need for scalable multi-pathogen testing platforms that can rapidly and accurately detect a broad spectrum of pathogens across diverse settings. A variety of platforms have been developed and used for AFI surveillance research, including TaqMan Array Cards (TACs) [3–7], BioFire [8, 9], and FastTrack Diagnostics [10]. Many AFI surveillance studies use multiple single-pathogen tests, which may be more time consuming and are limited in the number of targets. Surveillance researchers can also use serology to target antibodies, though the detection window may be more limited [11, 12].

The TaqMan Array platform has been used for the detection of multiple pathogens including bacteria, viruses, and parasites from a single sample. The ability to detect multiple pathogens across numerous samples, coupled with a standardized and reproducible format, makes TACs highly suitable for large-scale surveillance studies. In addition, the array platform is customizable, allowing for the use of study-specific assays that can be arrayed in a custom layout.

The Surveillance of Acute Febrile Illness Aetiology in Nigeria (SAFIAN) study aimed to detect 25 AFI-causing pathogens by using molecular assays to screen a population of acutely febrile patients in 2 distinct regions of Nigeria. The study was conducted at 2 tertiary hospitals, both equipped with on-site laboratories experienced in conducting polymerase chain reaction (PCR) testing. The University of Abuja Teaching Hospital (UATH) is located in Gwagwalada, a semiurban settlement in the Federal Capital Territory, and Irrua Specialist Teaching Hospital (ISTH) is located in Irrua, a rural settlement in Edo State. The research teams also gathered demographics, behavioral risk factors, and medical outcomes to describe the epidemiology of pathogens detected. This article describes the use of the TAC platform and its successful implementation at 2 clinical sites in Nigeria, highlighting challenges and key lessons learned from each step of the laboratory process—from preparation and start up to daily laboratory workflow and data quality control. Its goal is to provide information that will guide the facilitation of similar surveillance research using multi-pathogen molecular assay in other locations.

The national institutional review board (IRB) of Nigeria, National Health Research Ethics Committee of Nigeria (NHREC) reviewed and approved the study (NHREC/01/01/2077-08/03/2024C). The protocol was approved by the IRB of each hospital, ISTH and UATH. The RTI International IRB approved the reliance on NHREC for oversight as it satisfies the requirements of US Department of Health and Human Services regulations for the protection of human subjects (IRB registration no. IRB0000655). The protocol was reviewed and approved by the US Army Medical Research and Development Command, the Office of Human and Animal Research Oversight (OHARO), and the Office of Human Research Oversight (proposal log no. CT00030; award no. HDTRA1-22-1-0019; OHARO log no. E04891.1a).

## LABORATORY PREPARATION

Selecting the appropriate multi-pathogen detection platform was an important first step to achieve the study objectives. Key laboratory terms related to the choice of detection platform are defined in Table 1. Three commercially available multi-pathogen molecular platforms were considered (Table 2). The TAC platform provided the best solution for the SAFIAN study because it had preloaded cards, reducing the risk of human error and contamination, and was highly customizable. Neither the BioFire FilmArray nor FastTrack Diagnostics were customizable, and they had only a small number of relevant AFI pathogens for detection. Because the FastTrack assays were provided as a kit and not preloaded onto a testing platform, the assay setup would have been a manual process increasing the risk of human error and contamination.

**Table 1.** Definitions of Laboratory Terminology

Term	Definition
Multiplex assay	Ability to test for multiple targets (eg, different pathogens or genes) in a single reaction or well.
Multi-pathogen platform	Platform designed to detect or identify multiple pathogens (eg, bacteria, viruses, fungi, or parasites) simultaneously in a single sample.
Open/closed platform	Refers to whether a brand allows (open) or restricts (closed) the use of third-party tools, reagents, or software, with open platforms offering flexibility and closed platforms limiting users to proprietary components.
TNA extraction	Process to isolate TNAs—both DNA and RNA—from a biological sample for molecular testing.

Abbreviation: TNA, total nucleic acid.

**Table 2.** Commercially Available Multi-Pathogen Molecular Platforms for Pathogen Screening or Surveillance

Molecular Testing Platforms	Description	Benefits	Limitations
TAC (ThermoFisher)	Open customizable platform	Open platform to allow for customization; standardized and reproducible; multi-pathogen detection; multiplex capability; easy to use; no restrictions on targeting pathogens of interest.	High cost; requires separate TNA extraction step, increasing time, risk of human error, and risk of contamination; requires specific instruments, including PCR and centrifuge; research use only.
BioFire FilmArray (Biomerieux)	Closed, noncustomizable platform	Standardized and reproducible; multi-pathogen detection; no separate TNA extraction step; very easy to use.	High cost; closed platform not allowing for customization; detection restricted to panel-included pathogens; requires specific instrument; research use only.
FastTrack Diagnostics (Siemens Healthineers)	Assay kit	Multi-pathogen detection; multiplex assays; no special PCR instrument required.	Medium cost; not a testing platform with preloaded assays, increasing labor, time, risk of human error, and risk of contamination for manual test setup (ie, master mix, primers/probe, TNA); requires separate TNA extraction step, increasing time, labor, risk of human error, and risk of contamination; detection restricted to kit-included assays; research use only.

Abbreviations: PCR, polymerase chain reaction; TAC, TaqMan Array Card; TNA, total nucleic acid.

Although both site laboratories had existing PCR equipment, the TAC platform requires a compatible PCR thermocycler (QuantStudio 7 Pro; ThermoFisher) and centrifuge to accommodate a TAC plate. Thus, new equipment was procured, incurring a large expense (approximately $100 000 [USD]). The manufacturer provided installation and calibration services for the thermocycler and equipment training to the laboratory staff. The estimated cost per sample using our custom TAC platform was approximately $100 (USD), excluding reagent costs and equipment. Our SAFIAN team's in-country laboratory advisor provided technical assistance and guidance to the hospital laboratory staff during installation, training, and throughout the study.

While the customization of TACs was advantageous, it required the development of decision-making frameworks to select for the final list of pathogens for inclusion in the study [13, 14]. The final list is included in Table 3.

**Table 3.** List of Pathogen Targets on TaqMan Array Card Platform Designed for SAFIAN study

Pathogen Type	Target	Abbreviation
Bacterial	*Bartonella* spp.	BART
	*Brucella* spp.	BRUC
	*Coxiella burnetii*	CBUR
	*Leptospira* spp.	LEPT
	*Neisseria meningitidis*	NMEN
	*Rickettsia* spp.	RICK
	Pan-*Salmonella*	pSALM
	*Yersinia pestis*	YPES
Protozoan	*Leishmania* spp.	LEISH
	*Plasmodium* spp.	PLAS
	*Trypanosoma brucei*	TBRU
Viral	Crimean-Congo hemorrhagic fever virus	CCHFV
	Chikungunya virus	CHIKV
	Dengue virus	DENV
	Hepatitis E virus	HEV
	Hantavirus	HTNV
	Lassa virus	LASV
	Marburg/Ebola virus	MARV/EBOV
	Mpox virus	MPOX
	O'nyong'nyong virus	ONNV
	Pan-*Orthopoxvirus*	pOPX
	Rift Valley fever virus	RVFV
	West Nile virus	WNV
	Yellow fever virus	YFV
	Zika virus	ZIKV

**Figure 1.**
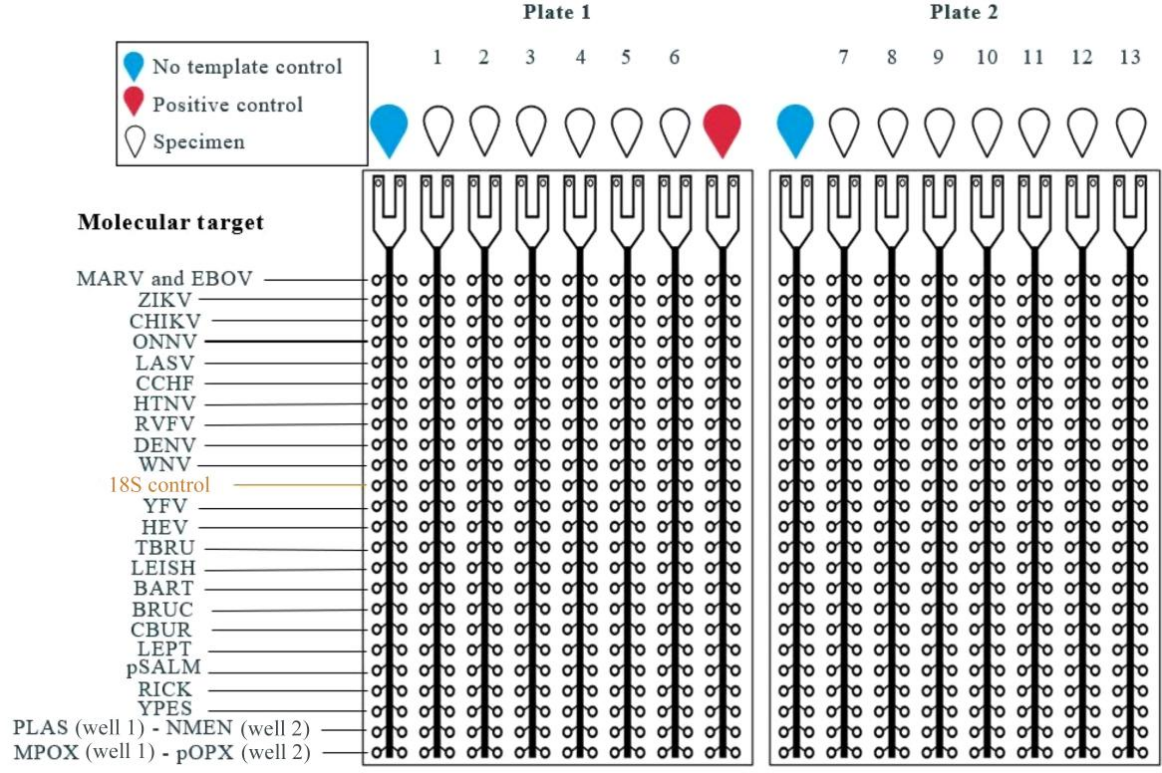
Custom layout of assays on the Surveillance of Acute Febrile Illness Aetiology in Nigeria (SAFIAN) TaqMan Array Card (TAC) platform. Plates 1 and 2 have the same molecular targets included in each row. See Table 3 for expansions of abbreviations for targets.

After the selection of pathogens was complete, we designed the array card layout (Figure 1) with assays selected or designed by the ThermoFisher team. ThermoFisher produced a custom positive control template containing all 26 targets (25 pathogens and 1 internal control). Of the 25 assays, 24 were commercially available, and 2 assays were designed by ThermoFisher. Because the manufacturing of these materials occurred in the United States, shipping time, shipping via cold chain, and customs clearance were important factors for consideration.

To prepare for the SAFIAN study, each laboratory designated space for allocation of samples for freezer storage, total nucleic acid extraction, TAC preparation, and execution of the TAC assay. Each of the designated spaces was outfitted with the appropriate equipment (eg, biocontainment chamber, centrifuge, incubator, micropipettes, and freezers) and a stable power supply to support continuous operation.

### Lessons Learned

The decision to implement the TAC platform aligned well with the study objectives, allowing the team to detect a diverse array of pathogens. A challenge encountered was a limitation with one of the assays. During the study, multiple participants had Lassa virus diagnosed using a hospital administered PCR test, per hospital protocol, but the TAC Lassa virus assay failed to detect the virus in these participants. Two participants who tested positive with the TAC assay did not test positive with the clinical PCR test. This raised concerns that the TAC assay was not designed to identify the local viral lineage. Unfortunately, due to the proprietary nature of all the ThermoFisher assays, encompassing both sequence and gene target information, we could not verify this limitation. However, we did further test all samples with the same single-pathogen PCR test used for clinical diagnosis (RealStar Lassa Virus RT-PCR kit; Altona Diagnostics). This single-pathogen PCR test covered lineages II–V. Therefore, we hypothesized that the TAC assay covered lineage I, which is not prevalent in Nigeria [15, 16].

For our surveillance study, because of restrictions to resource and scope, we did not validate the commercial assays before use, which we acknowledge is a study limitation. However, because surveillance aims to monitor population-level trends rather than provide patient-level diagnoses, the assays—designed against conserved genetic regions—are generally capable of detecting their intended targets. Although the use of commercially available TAC assays offers convenience, their performance should be validated for regional relevance—by referencing scientific literature, engaging local genomic experts, or confirming gene targets against circulating strains. Alternatively, time should be dedicated to selecting relevant assays that have been successful in previous studies. As demonstrated by the Lassa virus challenge, it is critical to know the strain-level variation when designing assays to accurately detect all clinically and epidemiologically relevant strains.

To strengthen the reliability of the TAC assays, a pilot testing phase and cross-platform validation are recommended, if feasible within the constraints of the study budget and scope. Before initiation of the full-scale study, pilot testing can be conducted using either locally archived samples with confirmed infection or simulated clinical samples to assess the suitability of the TAC assay and avoid gene target mismatches. Implementing a cross-platform validation by comparing a well-established regional assay with the TAC assay would enhance confidence in the performance and suitability of the TAC assay for regional deployment.

## LABORATORY WORKFLOW

**Figure 2.**
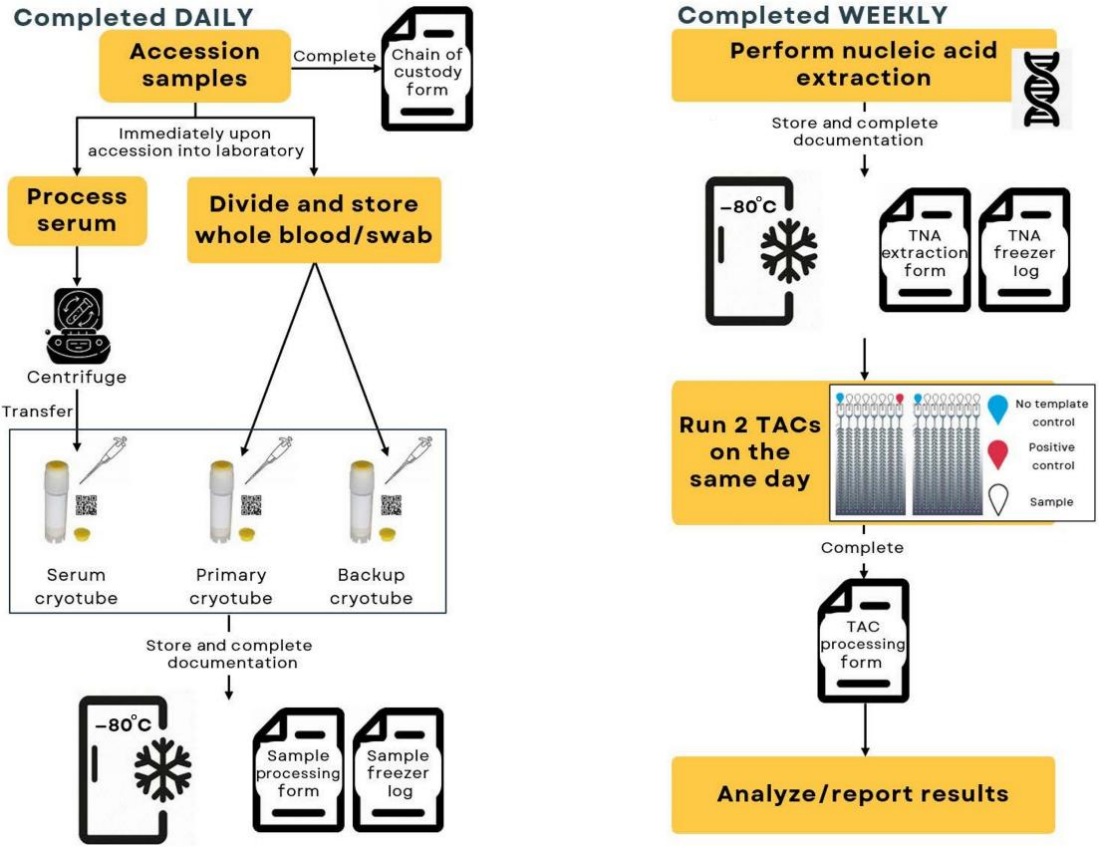
Laboratory workflow. Abbreviations: TAC, TaqMan Array Card; TNA, total nucleic acid.

Because the laboratory workflow (Figure 2) required for the TAC platform was new to the personnel and involved complex, multistep protocols, structured training and capacity building were essential to ensure accurate implementation and high-quality output. To help staff clearly understand the laboratory procedures, visual aids such as flow diagrams and other study documentation were provided to the sites, including standard operating procedures, a chain of custody form, job aids, a total nucleic acid extraction form, a TAC testing form, and sample freezer logs. The sites were provided with unique quick-response (QR)–coded sample labels, which were applied to the appropriate samples and corresponding documentation; this enabled linkage to participants and facilitated sample tracking from collection to TAC testing.

Specimen collection kits were assembled in advance. Study phlebotomists used one kit per participant, containing materials required for whole-blood, serum, and pox lesion specimen collection. Essential supplies consisted of gauze, gloves, a tourniquet, ethanol wipes, bandages, butterfly needles, a vacutainer ethylenediaminetetraacetic acid tube, a vacutainer serum tube, and a swab kit with universal transport medium. Each kit also contained a set of unique adhesive sample labels with QR codes that were placed on the appropriate sampling tubes and forms (eg, chain of custody form).

On-site training was conducted, covering every step of the workflow from sample collection to TAC analysis, and a validation test run was performed by each of the laboratory sites. Integral to the training were procedures to minimize exposure risk and preserve sample integrity. Following a successful validation run, the sites began conducting laboratory activities on participant samples.

### Lessons Learned

**Figure 3.**
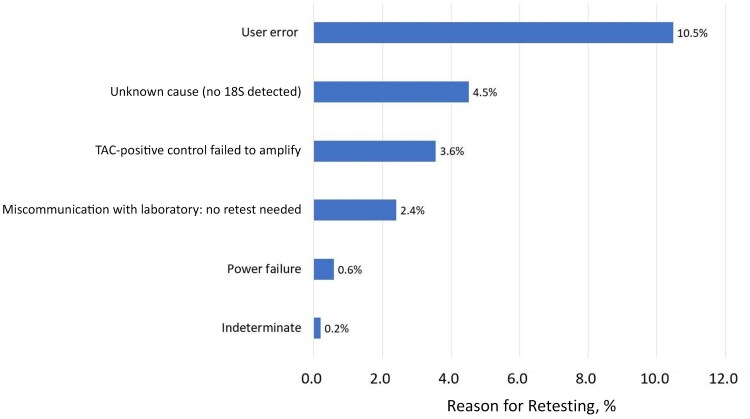
Reasons for TaqMan Array Card (TAC) retesting among all samples tested in the Surveillance of Acute Febrile Illness Aetiology in Nigeria (SAFIAN) study (N = 1041).

Overall, the study laboratory activities were conducted efficiently. However, there were a few issues, including unnecessary repeated testing of samples, delays in supply replenishment, and a contamination event. Figure 3 depicts the percentage of TAC retesting attributed to different causes; 226 of 1041 samples were retested (21.7%). The main cause for retesting, which occurred primarily at one laboratory site, was human error (10.5%) leading to contamination issues in the laboratory. This issue was addressed by implementing a thorough decontamination procedure of laboratory equipment and surfaces, enhancing personal protective equipment protocols to strengthen barriers against contamination (eg, double gloving and proper glove-to-sleeve coverage), and retraining staff to reinforce workflow practices that reduced the likelihood of cross-contamination (eg, organizing samples and materials so that hands and arms do not pass directly over them during handling). Unnecessary sample retesting (2.4%) ranked as the fourth most common reason for repeated testing. This procedural inefficiency and the challenge of supply chain delays were resolved by instituting structured oversight and routine communication. We implemented weekly laboratory meetings with the site laboratory teams, the SAFIAN study’s lead molecular microbiologist and the local logistics team to obtain updates on testing progress, any technical or logistic concerns, and any supply needs and status on supply orders.

Supply delays were further addressed with a range of supportive measures. These included enhancing storage infrastructure (eg, racking systems) and providing staff training to improve inventory management practices. Operational strategies were also adapted to ensure timely supply delivery, such as frequent follow-up meetings with international suppliers, periodic vendor sourcing for specialized laboratory items, and collaboration with a local shipping company to develop strategies to overcome logistical challenges such as poor road conditions. In addition, shared tracking documents were established and updated by the sites in real time. These tracking documents monitored supply orders from purchase to receipt, the TAC PCR test runs including assay verification (ie, whether the assay passed or failed), and samples that required retesting after final quality control review. All tracking documents were reviewed during the weekly meetings.

## INTERPRETATION OF TAC RESULTS

A preliminary analysis of TAC outputs was conducted onsite by each laboratory team, with quality control verification carried out by the SAFIAN study’s lead molecular microbiologist. A standard operating procedure was developed for the analysis process, which included instructions to manually set the threshold line for each of the 26 assays and guidelines for assessing TAC pass/fail outcomes, interpreting pathogen positivity, and identifying the need for sample retesting. The guidelines for TAC pass/fail outcomes and for sample retesting were defined based on the criteria established from the controls (negative, positive, and internal 18S). For example, the entire array card failed if ≥3 assays for the negative control amplified or if ≥3 assays for the positive control failed to amplify.

All samples on the failed card were retested. Samples on array cards that passed were retested if the internal 18S control did not amplify or if 1 or 2 assays failed for the negative or positive controls. For the latter occurrence, only samples that showed no amplification for the same failed assays in the positive control or showed amplification for the same failed assays in the negative control were retested. For pathogen detection, 22 of the TAC assays were run in duplicate, and 4 were run as singlets. If the singlet assays yielded an amplification curve, this was considered positive for the target pathogen. For the duplicate assays, a pathogen was considered positive if ≥1 of the duplicate runs amplified. The duplicate assays provided better sensitivity especially for low-yield pathogens. All final positive and negative results were integrated using R Studio software, version 2023.06.0.

### Lessons Learned

Most of the TAC assays yielded distinctly positive and negative results, characterized by the presence or absence of a strong, well-defined amplification curve. However, there were some assays which provided curves that were not as well defined. Due to the constraints of this surveillance study, retesting was limited, necessitating the direct and careful interpretation of assays for positivity and negativity. Although this was relatively subjective, less-clear PCR curves were assessed by expert evaluation of the curves’ visual characteristics in both linear and logarithmic plots. The primary criterion for assessment was the presence of a classic sigmoidal amplification curve in the linear plot, with emphasis on identifying a defined exponential phase in both the linear and log-scale views. If the curves met these criteria, then the assay was interpreted as positive for the specific pathogen target. The SAFIAN study’s lead molecular microbiologist provided final review and interpretation of all TAC results, ensuring a standardized assessment process.

Most of the assays on the array card were arranged as duplicates enhancing the likelihood of pathogen detection by mitigating issues such as stochastic variation, technical variability (eg, pipetting errors or uneven sample distribution), and low target copy numbers. To maximize the number of assays on the array cards, we made the strategic decision to run 4 assays (PLAS [*Plasmodium* spp.], NMEN [*Neisseria meningitidis*], MPOX [mpox virus], and pOPX [pan-*Orthopoxvirus*]) as singlets rather than duplicates. *Plasmodium* spp., endemic and widespread in Nigeria [17, 18], lead to infections typically characterized by high parasitemia [19]; thus, we anticipated that the pathogen would be readily detectable by a single assay.

*N. meningitidis* has been shown to be of public health concern in Nigeria, with a severe epidemic in 1996 [20] and an outbreak of disease from October 2022 to April 2023 [21]. However, this pathogen was not a high priority for AFI, since the most recent outbreak (2022–2023) did not affect the 2 study sites and meningococcal disease can present with distinguishable symptoms (eg, stiff neck), inconsistent with the nonspecific case definition of AFI. Like malaria, meningococcal infections typically manifest with high bacteremia, and a single assay for detection would be sufficient. Although the assays for mpox virus and pan-*Orthopoxvirus* were performed as singlets, the pOPX assay offered redundancy in mpox virus detection due to its inclusion in the *Orthopoxvirus* genus; furthermore, its detection capability included other members of the genus.

## CORE LESSONS AND CONCLUSIONS

The primary insights that could guide successful implementation of the TAC platform in other low-resource settings, based on experience from the SAFIAN study, are shown in Table 4. Careful consideration of these key factors in the design of a large surveillance study will facilitate more accurate timelines and efficient laboratory workflows with fewer errors, resulting in improved efficiencies and standardized, high-quality results. This approach also promotes collaboration and shared ownership among the study team.

**Table 4.** Key Factors for Successful Implementation of TaqMan Array Cards

Critical Factors	Primary Considerations
Procurement timeline	This process can be lengthy with unexpected delays, especially for items not available for local purchase; foreign shipments may require specific documentation (eg, import documents or waivers) needing approval, adding to the timeline.
Assay selection and validation	Commercial assays should be validated or time should be dedicated to identifying and selecting robust assays for pathogen detection.
In-person training and mentorship	Laboratory staff should observe each step of the workflow demonstrated by an experienced molecular microbiologist, particularly when handling infectious samples; in turn, the microbiologist should observe staff and provide targeted and constructive feedback.
Regular meetings	Recurring meetings should be held between the laboratory site teams and in-country collaborators to provide status updates and address concerns such as supply needs, contamination, and testing failures.
Study lead scientist	This role involves overseeing all laboratory operations and data analysis, including the final quality control of TAC results and their interpretation, to standardize the approach for consistent negative and positive determinations.

Abbreviation: TAC, TaqMan Array Card.

Conducting multi-pathogen surveillance research is critical for identifying the diverse causes of AFI, offering valuable insights to inform clinical diagnosis and public health strategies. This effort can be significantly strengthened by efficient laboratory detection tools, such as the TAC platform. With the expansion of laboratory technologies available globally, research investigators should implement strategies for improving research quality and maximizing outcomes. The use of TACs presents specific operational considerations that investigators should be aware of before study initiation. Gaining a clear understanding of these methodological nuances in advance can help new users anticipate potential challenges, streamline implementation, and enhance the overall quality and efficiency of their research. Furthermore, the TAC platform should be evaluated as a sustainable surveillance tool that is feasible in low- and middle-income countries following its initial setup and implementation in a research study.

Implementing the TAC platform for multi-pathogen surveillance research in low-resource countries such as Nigeria requires diligent and meticulous oversight and collaborative coordination. Laboratory staff must manage multiple overlapping tasks that require consistent management, and the active coordination of these tasks is a shared responsibility, especially at the site level, to achieve successful execution.
